# The Characterization of Deqi during Moxibustion in Stroke Rats

**DOI:** 10.1155/2013/140581

**Published:** 2013-09-08

**Authors:** Zhimai Lv, Zhongyong Liu, Dandan Huang, Rixin Chen, Dingyi Xie

**Affiliations:** ^1^The First Affiliated Hospital of Gannan Medical University, Ganzhou, Jiangxi Province 341000, China; ^2^Affiliated Hospital of Jiangxi University of TCM, Nanchang, Jiangxi Province 330006, China; ^3^Gannan Medical University, Ganzhou, Jiangxi Province 341000, China

## Abstract

The efficacy of acupuncture and moxibustion is closely related to Deqi phenomenons, which are some subjective feelings. However, no one has reported the objective characterization of Deqi. Our preliminary research has found a phenomenon of tail temperature increasing (TTI) obviously in some stroke rats by suspended moxibustion at the acupoint dà zhuī (DU 14), which is similar to one characterization of Deqi during moxibustion that moxibustion heat is transferred from the original moxibustion acupoint to the other areas of the body. We wonder whether TTI is the objective indicator of Deqi characterization in animals. The present study showed that the stroke rat's recovery was also associated with TTI phenomenon. This suggests that TTI phenomenon is one objective characterization of the Deqi in stroke rats. Application of the TTI phenomenon contributes to explore the physiological mechanism of Deqi.

## 1. Introduction

It is considered that the clinical efficacy of acupuncture and moxibustion is closely related to Deqi phenomenon in traditional Chinese medicine. The characterizations of the Deqi during acupuncture treatment have been elaborated in acupuncture textbooks, which are some subjective feelings, such as soreness and heaviness [1]. Meanwhile, the characterizations of the Deqi during moxibustion treatment have also been clarified in clinical heat-sensitive moxibustion practice over the past decade [2], including the following: (a) moxibustion heat penetrates deep into the tissues or internal organs of the body; (b) moxibustion heat is transferred from the original moxibustion acupoint to the other areas of the body; (c) moxibustion heat could elicit other sensations, including pressure, soreness, heaviness, and dull pain, at the surface of the skin or deep tissues. However, the above characterizations are also some subjective feelings. The objective characterization of Deqi during moxibustion has not been reported yet. Our preliminary research has found a phenomenon of tail temperature increasing (TTI) obviously in some stroke rats by suspended moxibustion (SM) at the acupoint dà zhuī (DU 14), which is similar to the characterization (b) of Deqi during moxibustion in humans [3]. We hereby propose the hypothesis that TTI is the objective indicator of Deqi characterization in stroke rats during moxibustion. The present study was designed to verify this hypothesis.

## 2. Methods

### 2.1. Animal Preparation

A total of 75 adult male Sprague-Dawley rats (220 to 250 g) were used in the experiment. The rats were maintained in a cage at room temperature 23 ± 2°C, with controlled humidity 60 ± 5% and 12-hour day/night cycle, with a maximum of five rats per cage. Firstly, 30 rats were divided randomly into 2 groups: (1) Sham operation with SM for 60 min group (sham, *n* = 10) and (2) ischemia with SM for 60 min group (M, *n* = 20). They were all treated for 3 days. According to the tail temperature change, the M group was further divided into two subgroups, including a nonincreasing subgroup (≤1°C an average of 3 days, non-TTI group) and an increasing subgroup (>1°C an average of 3 days, TTI group). Then, four points around the rat's torso were heated in five TTI rats. Secondly, another 45 rats with transient middle cerebral artery occlusion (tMCAO) operation were divided randomly into 2 groups: (1) ischemic control group (C, *n* = 15) (2) ischemia with SM for 60 min group (M60, *n* = 30). Rats in M60 group were treated with SM for 7 days. Like the first part of the experiment, the M60 group was also further divided into two subgroups, respectively, including the M60-non-TTI subgroup and the M60-TTI subgroup. All experimental procedures involving the use of animals were conducted in accordance with NIH Guidelines and approved by the Animal Use and Care Committee for Jiangxi University of TCM.

### 2.2. Preparation of Experimental Stroke Model in Rats

The rats were anesthetized with an intraperitoneal injection of sodium pentobarbital (3%) at a dose of 30 mg/kg. Core body temperature was monitored using a rectal probe and maintained at 37 ± 0.5°C by a heating lamp and a heating pad. The middle cerebral artery occlusion was achieved by the Intraluminal Filament method as previously described [4]. After 2 h of occlusion, the fishing line advanced to the origin of the middle cerebral artery was withdrawn to allow for reperfusion. Sham-operated rats were manipulated in the same way, but the MCA was not occluded. Adequacy of vascular occlusion and reperfusion was assessed by Laser Doppler Monitoring (PeriFlux 5000, Perimed AB, Stockholm, Sweden) of cerebral cortical perfusion. Regional cerebral blood flow in the middle cerebral artery territory was reduced to <20% of baseline, after advancing the fishing line to the origin of the MCA, and reconstituted to >60% of baseline after removal of the fishing line. Rats dying within 24 hours after surgery or displaying a neurological score of 0 were excluded from the final analysis.

### 2.3. Suspended Moxibustion

A special cage in which the rat can maintain a comfortable position and the rat's motion is restricted was used while testing. The cage was convenient to the operation of SM. Room temperature was maintained at 25 ± 2°C for the entire experimental process. In the first part of the experiment, DU 14, which is considered very important for brain functions [5], was heated by SM using a moxa (exclusively used on animals, length 12 cm, diameter 0.6 cm, made by the Affiliated Hospital of Jiangxi University of TCM, China). Then, five rats, selected randomly from the TTI group, received SM operation at four points. The first point was DU 14. The second point was located at the one that was 2 centimeters right beside the acupoint of DU 14. Both of them were heated at approximately 3 cm high over the hairless skin. The third point was located at the extension line of the longitudinal axis of the rat. The fourth point was located at perpendicular of the tail's midpoint. The third and fourth points both had the same distance far from rat's tail midpoint, which was identical to the distance between the acupoint of DU 14 and rat's tail midpoint. In the second part of the experiment, the heating point was also the acupoint of DU 14.

### 2.4. Tail Temperature Measurement

The rats' midpoint tail temperature was recorded once every 2 minutes precisely by an electrodigital thermometer (Shanghai Medical Instrument Factory, Shanghai, China) in process of SM treatment. The testing environment was kept quiet, and the room temperature was maintained at 25 ± 2°C. The rats were placed in a cage for 30 min before the experiment started.

### 2.5. Neurological Assessment

Neurological assessment was performed at 0, 1, 3, and 7 days after transient middle cerebral artery occlusion by a researcher who was unaware of the experimental groups, using a modified neurological severity score, which were graded on a scale of 0 to 18 (normal score, 0; maximal deficit score, 18), as previously described [6].

### 2.6. Statistical Analysis

Data was analyzed using one-way analysis of variance (ANOVA) with post hoc Newman-Keuls multiple-range test for multiple groups. The pearson correlation coefficient was also calculated between the neurological deficits score and change of tail temperature. SPSS 10.0 was used for analysis. *P* < 0.05 was considered statistically significant. All values were expressed as the mean ± SD.

## 3. Results

### 3.1. Quantitative Analysis of Experimental Animals

In the first part of the experiment, 2 of the 20 ischemic rats (1 death and 1 displaying 0 score) met at least one of the exclusion criteria. 9 ischemic rats exhibited TTI, and 9 subjects showed non-TTI. In the second part, 2 of the 15 rats in C group (2 death) and 3 of the 30 rats in M60 group (2 death and 1 displaying 0 score) met at least one of the exclusion criteria. There were 13 subjects in M60-non-TTI subgroup and 14 subjects in M60-TTI subgroup. Thus, 28 rats of the first part and 40 rats of the second part were included in the final analysis.

### 3.2. Tail Temperature Change following Suspended Moxibustion

**Figure 1:**
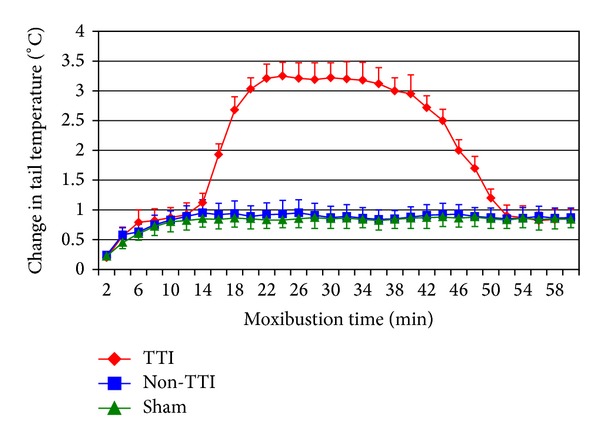
Change in tail temperature induced by SM in the first part of the experiment. Because the change of tail temperature was similar among the three consecutive testing days, data of the first day were presented as a representative. Data were expressed as mean ± SD.

**Figure 2:**
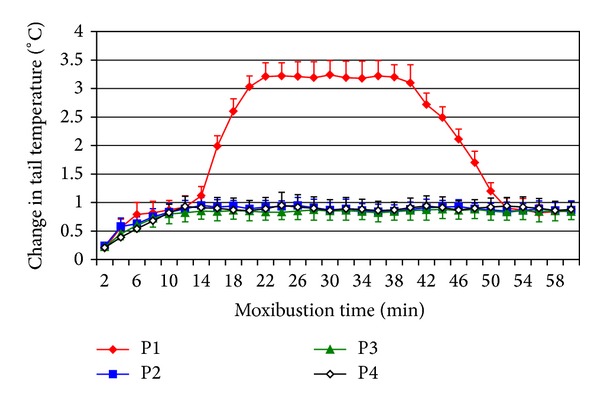
Change in tail temperature induced by SM at four points, respectively, around TTI rat's torso. Data were expressed as mean ± SD. P1: first point; P2: second point; P3: third point; P4: fourth point.

In the first part of the experiment, tail temperature began to quickly increase immediately after suspended moxibustion. At about 5–10 min, the temperature reached a relatively stable level but less than 1°C on average. 9 tMCAO rats, as well as the sham rats, maintained this level (non-TTI) throughout the treatment session. However, the other 9 tMCAO rats exhibited TTI (more than 2°C on average). Furthermore, the tail temperature in the rats with TTI increased to a peak value at around 15 min, and the peak temperature was maintained until 40 min, at which a decline began to appear. At about 50 min, the tail temperature decreased to a level similar to that of the non-TTI or sham rats. The results were similar during the 3 consecutive days (Figure 1). Five stroke rats were randomly selected from TTI group and received SM operation for 60 min at four points, respectively. The tail temperature exhibited TTI by heating the first point. However, heating the other three points did not elicit TTI (Figure 2). 

### 3.3. Neurological Deficits Score

**Figure 3:**
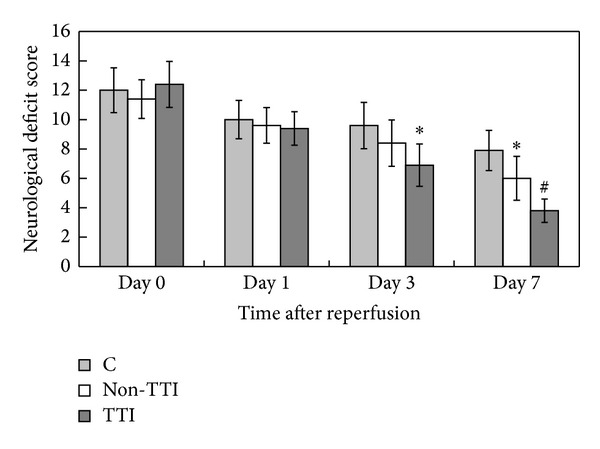
SM on neurological deficit score in the tMCAO rats. Data were presented as mean ± SD. *P < 0.05 versus C group; ^#^ *P* < 0.05 versus C and M60-non-TTI groups using one-way analyses of variance.

To investigate the efficacy of SM for 60 min with TTI, we examined the neurological deficit score of tMCAO rats in the second part of the experiment. The results revealed that the M60-TTI group significantly reduced neurological deficit score at 3 days after reperfusion, compared with the C group (*P* < 0.05). This group further ameliorated the neurological deficit score at 7 days after reperfusion, compared with the C and M60-non-TTI (*P* < 0.05) groups. The M60-non-TTI group reduced neurological deficits score markedly at 7 days compared to the C group (*P* < 0.05) (Figure 3). 

### 3.4. Behavior Correlation with Tail Temperature Increase

**Figure 4:**
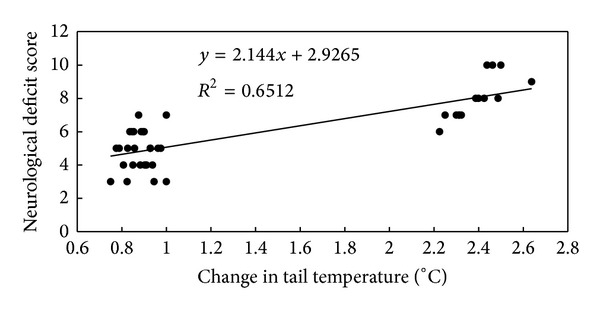
The change of tail temperature increase correlated with the neurological deficits score. *r* = 0.807, *P* < 0.01.

In order to explore the relationship between the reduction of neurological deficits score and the change of tail temperature increase induced by SM, we calculated the intersubject Pearson correlation coefficient between both mentioned above. We found that the reduction of neurological deficits score was positively correlated with the change of tail temperature increase induced by SM (*R* = 0.807, *P* < 0.01) (Figure 4).

## 4. Discussion

Deqi is a composite of unique sensations that is produced during acupuncture or moxibustion stimulation. We have paid attention to the clinical characterizations of Deqi during moxibustion for 20 years and summarized its characterizations as previously described. Furthermore, we have confirmed the efficacy of SM with Deqi is superior to that without Deqi in clinic [2, 7–9]. Further investigation of the biological mechanism of Deqi during moxibustion depends on establishing the objective assessment of Deqi characterization, especially in animal study. However, few investigators have reported the objective characterization of Deqi in animals. In our previous study, we have accidentally found a TTI phenomenon in some stroke rats by SM at the DU 14 [10]. Based on the TTI phenomenon similar to one of Deqi characterizations during SM in clinic, we wonder whether TTI is the objective indicator of Deqi characterization in animals. 

In this study, we have observed the change rule of TTI induced by SM at the DU 14 of tMCAO rats. The tail temperature in the rats with TTI increased beyond other subjects at about 15 min, and the peak temperature was maintained until 40 min, at which the decline began to appear. At about 50 min, the tail temperature decreased to a level similar to that of the non-TTI or sham rats. The results also suggested that TTI did not appear in sham-operated rats. This indicates that TTI is relevant to the model of stroke. It is consistent with the fact in clinic that Deqi phenomenon during SM is highly relevant to the morbid condition of human body [2, 11]. 

The tail is far from the heating acupoint. How is the TTI induced? In order to exclude the impacts of the conductive (the second point) and radiant (the third and fourth points) heat on TTI generation, we set three other heating points except DU 14 as comparisons. The results showed that heating on three other heating points, respectively, exhibited no TTI. This proclaimed that conductive heat and radiant heat were not the reasons of inducing TTI phenomenon. However, in traditional Chinese channel theory, DU 14 and the tail are both on the DU channel (Governor Vessel) [5]. Stimulating the DU14 with SM could provide heat for the tail through the channel. The results of neurological deficit score further revealed that the tMCAO rats with TTI during SM recovered better than those without. This confirmed that the efficacy of SM was closely related to TTI phenomenon. It is also consistent with the fact in clinic that clinical efficacy of moxibustion is closely related to Deqi phenomenon. From this perspective, TTI could be considered as a characterization of Deqi in tMCAO rats during SM. 

Why did not the other 13 tMCAO rats exhibit TTI? It is similar to the observation in clinic that acupoints that can be stimulated to cause Deqi phenomenon may have different locations in subjects who are afflicted with the same disease [2]. In the present study, the point (DU 14) for SM treatment was fixed. Therefore, some of them exhibited TTI, while others did not. Acupoints other than DU 14 were stimulated in the 13 tMCAO rats in order to produce TTI. As the occurrence rate of TTI is 40–60% in our previous study [3], the number of moxibustion-treated groups we designed is twice of the other groups (such as sham or C group) so that the number of non-TTI or TTI rats selected from the total tMCAO rats is almost the same as the sham or C group.

In conclusion, this study reported a TTI phenomenon in tMCAO rats with SM and proved this phenomenon was associated with the tMCAO rat's recovery. There was enough reason to believe that TTI phenomenon was one of the Deqi characterizations in tMCAO rat. Application of the TTI phenomenon contributes to explore the physiological mechanism of Deqi. 

## Abbreviations

SM: Suspended moxibustion
TTI: Tail temperature increase
tMCAO: Transient middle cerebral artery occlusion.
